# Tissues as networks of cells: towards generative rules of complex organ development

**DOI:** 10.1098/rsif.2023.0115

**Published:** 2023-07-26

**Authors:** Sabine C. Fischer, George W. Bassel, Philip Kollmannsberger

**Affiliations:** ^1^ Center for Computational and Theoretical Biology, Faculty of Biology, University of Würzburg, Würzburg, Germany; ^2^ School of Life Sciences, The University of Warwick, Coventry, UK; ^3^ Biomedical Physics, Department of Physics, Heinrich Heine University Düsseldorf, Düsseldorf, Germany

**Keywords:** developmental biology, complex networks, cell graphs, connectivity, generative models

## Abstract

Network analysis is a well-known and powerful tool in molecular biology. More recently, it has been introduced in developmental biology. Tissues can be readily translated into spatial networks such that cells are represented by nodes and intercellular connections by edges. This discretization of cellular organization enables mathematical approaches rooted in network science to be applied towards the understanding of tissue structure and function. Here, we describe how such tissue abstractions can enable the principles that underpin tissue formation and function to be uncovered. We provide an introduction into biologically relevant network measures, then present an overview of different areas of developmental biology where these approaches have been applied. We then summarize the general developmental rules underpinning tissue topology generation. Finally, we discuss how generative models can help to link the developmental rule back to the tissue topologies. Our collection of results points at general mechanisms as to how local developmental rules can give rise to observed topological properties in multicellular systems.

## 1. Introduction

Developmental biology has historically been limited to being a principally qualitative discipline, where patterns were observed and compared, but rarely quantified [1]. Applying quantitative approaches is increasingly proving to be powerful to characterize patterns and morphology in tissues [2,3]. The developmental rules underpinning pattern formation in tissues in general are well-known [4,5]. However, the links between specific processes and the resulting patterns are only beginning to be understood due to the non-intuitive nature of how genes lead to emergent phenotypes [6]. In this context, mathematical and modelling approaches are of value.

**Figure 1.**
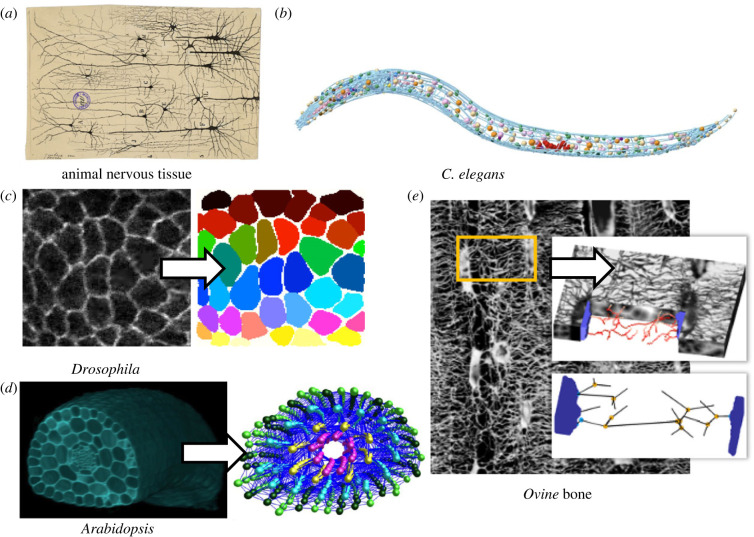
Examples of network analysis applied to developmental systems. (*a*) Cajal diagram describing neuronal connectivity (Museo Cajal, Madrid, Spain). (*b*) *Caenorhabditis elegans* network describing the connectivity between neurons, from [10] (CC-BY). (*c*) A confocal image of the *Drosophila* epithelium and an illustration as to how it can be segmented (original work). (*d*) Three-dimensional confocal image stack of a plant hypocotyl and its abstraction into a network describing cell connectivity, adapted from [11]. (*e*) Osteocyte network in bone and image analysis quantifying its connectivity within the bone tissue, adapted from [12].

Network approaches have been used extensively to study molecular interactions within cells. A large number of measures enables the analysis of these networks to understand system function [7]. Many of these same methods and analyses can also be applied to additional scales of biological complexity, including the study of cell interactions in tissues [8,9]. A perspective of multicellular systems as interacting collectives of cells can be traced back to the work of Ramon y Cajal and his examination of mammalian nervous systems (figure 1*a*) [13]. The creation of these original ‘wiring diagrams’ of nervous connectivity by looking through a microscope and drawing led to a step change in our conception as to how collections of cells come together to generate emergent tissue function [14].

The abstraction of cells into nodes and their physical associations as the edges led to the formal description of tissues as network graphs which could be topologically analysed. The systematic mapping of the *Caenorhabditis elegans* nervous system, and its subsequent analysis using network science, enabled the prediction of the function of individual neurons within this complex tissue system [14] (figure 1*b*). These studies were instrumental in characterizing the organization of cells in tissues, and in showing that structure–function relationships scale to cellular architecture in nervous systems [15]. The abstraction of neuronal organization into networks provides a means to approach these questions in discrete terms using network science, providing a quantitative path towards understanding system function following mathematical principles [16]. In this way, the manner by which cell organization shapes and constrains organ function can be addressed. An understanding of cell topology may therefore provide insight into emergent tissue function.

While the original transformations of tissues into networks of cells have come from studies involving nervous systems, more recently, these same approaches are being applied to a diverse set of non-neuronal developmental systems, including plant tissue [11], fly wing epithelia [8], human bone [17], or snowflake yeast [18], examples of which are shown in (figure 1*c–e*). In this review, we start by first introducing biologically relevant network measures and analysis methods in order to set the basis for understanding the subsequent sections. We then highlight a few examples from different areas of developmental biology where these approaches have been applied. Finally, we summarize the general developmental rules underpinning tissue topology generation and discuss how generative models can help to link the developmental rule back to the tissue topologies.

## 2. Tissues as networks of cells

Networks representing the connectivity of cells can be viewed in several ways. *Structural networks* describe the physical associations between cells [19]. This is akin to a road map which captures all the possible routes cars can travel upon. In tissue terms, this describes the potential communication between cells. *Functional networks* describe the observed communication and information flow between cells in tissues, akin to where traffic is observed in a road network [20]. Functional networks are constrained by physical networks, and are dynamic in the sense that intercellular communication can be modulated across development. In this review, networks of cells refer to structural networks. The following section describes how structural networks of cells can be extracted from data and quantified and introduces the relevant terminology. Despite being rather technical, this forms the basis for the applications and modelling approaches discussed in the subsequent parts.

The architecture of structural networks is generally obtained by imaging and subsequent analysis. The modality of imaging is not restricted to a specific microscopy technique and depends on the type of network to be analysed. Serial transmission electron microscopy has been used to map the first complete connectome in *C. elegans*. Other approaches such as whole mount three-dimensional microscopy have also proved useful towards the construction of tissue connectomes [21].

The images are further processed by cellular segmentation and network generation [12,22]. In structural cell networks, nodes represent cells and edges represent the connections between cells. In the most simple case, an edge between two vertices is included in a network if two cells are physically connected. This can be established from cell boundary information in the images. In the absence of a cell boundary marker, nuclei may be imaged and edges established if the distance between two cells is smaller than a given threshold [21]. This threshold represents a typical distance of communication that can be assumed in the respective tissue. A number of open-source tools exist that implement the generation of cell graphs from tissue images [23,24].

The inclusion of multiple fluorescent reporters in tissue images can provide additional dimensions of data to extracted networks. Nodes can be weighted using geometric features, such as cell size [25], or according to different molecular states such as the expression of genes, proteins [26] or biosensors (examples provided below). Edges can also be weighted by geometric features such as intercellular interface size, or molecular determinants such as the abundance of transporters or observed molecular fluxes between cells.

**Figure 2.**
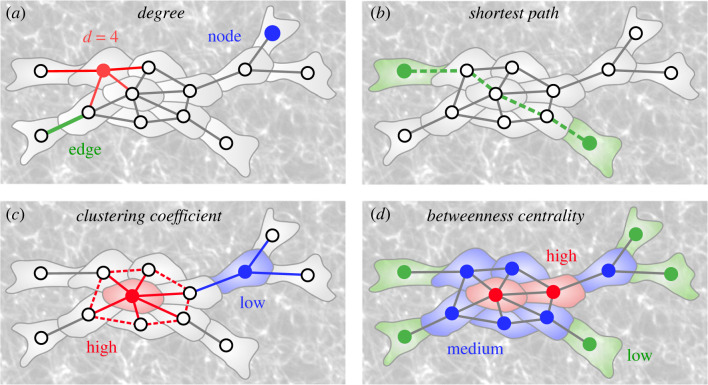
Illustrations of biologically relevant network measures. (*a*) Node degree is the number of edges (direct neighbours) connected to a node (cell). (*b*) The topological shortest path between two nodes (cells) is the minimum number of nodes (cells) between one node (cell) and another. (*c*) The clustering coefficient of a node (cell) is the fraction of possible edges between its direct neighbours that actually exist. (*d*) The betweenness centrality of a node (cell) is the fraction of all shortest paths in the network (tissue) that run through that node (cell).

Graph theory provides a plethora of measures to analyse network topologies [27]. In the context of tissues, many of these can provide biologically relevant information. The simplest such measure is the number of neighbours a cell has (figure 2*a*) [28]. This is termed the *degree*, and provides a local measure of how potentially influential a cell is within its immediate context [29]. Higher-order properties of tissues may also be examined. A commonly used approach to understand the significance of a node within a transportation network, such as a multicellular tissue, is to examine the path length a cell lies upon. Following a *shortest path* represents an optimized mode of travel (figure 2*b*). Network measures have been developed which identify nodes that lie upon shortest paths. Such nodes represent cells which are topologically positioned for optimized transport and communication. They also mediate the movement of information across networks, providing an important function in controlling tissue-wide communication.

If prior knowledge of the complete network is known, *betweenness centrality* can be used to find nodes which lie upon shortest paths (figure 2*d*) [28]. In the absence of complete knowledge of network topology, as is the case in biological tissues, *random walk centrality* can be used to identify nodes on shortest paths through the use of random walkers and the frequency they pass through nodes on their way from one cell to the next. A variation on this, termed *navigation centrality*, makes use of local network knowledge while following a gradient, for example a morphogen in the case of a tissue, to identify near-optimal shortest paths through tissues [30].

The higher-order efficiency of transport across multicellular networks can also be examined using the *local* and *global efficiency* measures [31]. *Local efficiency* examines the impact the random failure of nodes has on increasing the number of shortest paths between pairs of nodes within the vicinity of the failure. The resilience of the network to random errors can be measured with respect to the increased costs of transportation locally. *Global efficiency* examines the efficiency of routing of information across the whole network. This is calculated by considering the shortest paths between all pairs of nodes, and addresses how easy information can move across a given network. A trade-off between communication efficiencies at each local and global scales is therefore present, and provides a biologically relevant means to explore spatially constrained transport networks such as multicellular organs.

## 3. Examples in different organisms

In the following section, we highlight a number of examples from the literature where spatial cell networks were measured and quantified in diverse biological systems ranging from fly wing epithelia and plant tissue to mouse embryos, cancer cells as well as neurons and human bone.

### 3.1. Topology of *Drosophila* epithelia

**Figure 3.**
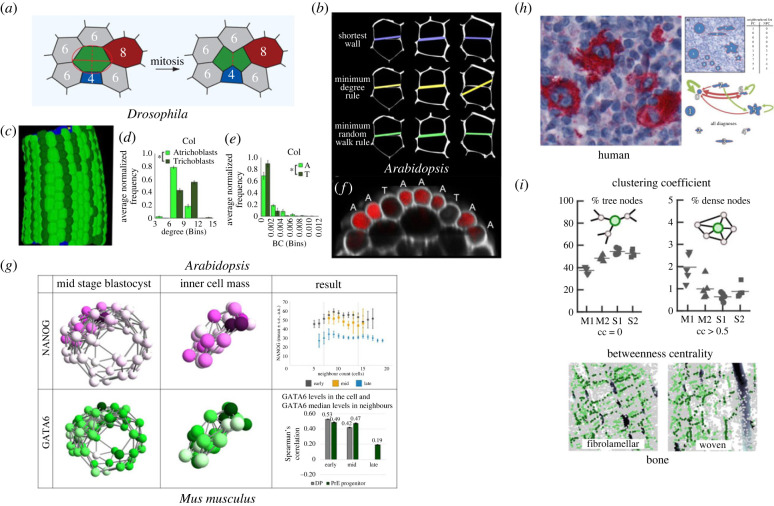
Examples of the application of network approaches towards understanding tissue organization and function. (*a*) Local cell division orientation in the *Drosophila* epithelium follows rules based on the constraint of degree across the tissue. (*b*) Orientation of cell division planes in the *Arabidopsis* shoot apical meristem conforms to local geometric (shortest wall), local topological (minimum degree) and global topological (minimum random walk) rules, adapted from [34]. (*c*) Three-dimensional segmentation of the cells of the *Arabidopsis* hypocotyl with two epidermal cell types (trichoblast and atrichoblast) given distinct colours, adapted from [11]. (*d*) Degree distribution of each hypocotyl epidermal cell type, adapted from [11]. (*e*) Betweenness centrality distribution of each hypocotyl epidermal cell type, adapted from [11]. (*f*) Confocal image of the hypocotyl showing preferential movement of fluorescein through atrichoblast cells, adapted from [11]. (*g*) Network analyses of the inner cell mass in mouse embryos reveals local patterns in early and mid blastocysts, adapted from [25]. (*h*) Hodgkin lymphoma cells exhibit preferences for the shape of their neighbouring cells, from [26]. (*i*) The osteocyte network in highly organized fibrolamellar bone shows a more tree-like topology (top) and aligned paths in maps of betweenness centrality (bottom) compared with disordered woven bone, adapted from [17].

The development of the *Drosophila* wing disc epithelia has been examined using network-based approaches. Local topological examination of structural cell networks found their local neighbourhood (degree) to be focused on six adjacent cells [8,32]. This observation quantified a previously unknown topological property of the epithelial tissue that shows an overall consistency across different tissues from different animals as well as the plant epidermis [8,32,33]. To conserve the establishment of six neighbours, cell division planes are biased according to neighbouring cell connectivity (figure 3*a*). In this way, local division rules lead to complex emergent tissue properties. To decipher morphogenetic mechanisms, it is, however, interesting to investigate differences between epithelia from different regions or developmental stages of *Drosophila* and to compare those with epithelia from other species. In this regard, refined analyses with more complex network measurements have been very valuable [35–37]. The implementation of a feature vector that contains the cell area, node degree, the clustering coefficient and the average degree of neighbours has allowed comparing *Drosophila* prepupal wing and notum as well as larval wing epithelium [35]. Application of discriminant analysis has indicated differences between the two wing tissues and the two prepupal tissues. Comparing *Drosophila* and chick epithelia, a very pronounced separation occurred. Interestingly, for the interspecies comparisons, the geometric feature cell area was much less informative than the three network measures. The feature vector approach has later been extended to include 40 features of three types: geometric features related to the size and shape of a cell, network characteristics of the cells such as node degree and network characteristics of the images such as clustering coefficient and betweenness centrality [36]. Principal component analysis of the extended feature vectors resulted in a clear separation between third instar *Drosophila* wings discs and early prepupa wing discs, a result that was not clear from a mere analysis of the distributions of the number of neighbours in these tissues.

### 3.2. Cellular organization and morphogenesis in plants

A similar study to that done on the *Drosophila* wing disc was undertaken in the apical stem cell niche of plants called the shoot apical meristem (SAM). Here, the higher-order properties of the cell network (cells as nodes and edges their physical associations) were examined, looking at both the local property of degree and higher-order property of path length. This was possible based on the complete capture of the system in three dimensions, in contrast to local sampling.

The organization of cells in the SAM was found to be optimized for maximal path length [34]. In other words, no topological shortcuts are present in the tissue and it takes an equally long number of maximal steps in each direction to traverse the tissue. In instances where cells lay upon shorter paths across the tissue, these same cells had a greater propensity to divide. Furthermore, the plane of division in these low-path-length cells was oriented such that the two daughter cells each lay upon maximal path lengths (figure 3*b*). It was therefore possible to use the higher-order property of path length to both predict which cell will divide next, and predict the orientation of the division plane that is followed.

The absence of a topological bias in the SAM was hypothesized to play a functional role in the positioning of new organ primordia which emerge in its flanks. The hormone auxin is dynamically transported to different positions across the SAM following the golden angle [38]. The topological uniformity of the SAM allows for this dynamic process to unfold equally in all directions, enabling robust organ positioning. The use of a mutant with altered cell shapes and tissue path length showed a decrease in the robustness of organ positioning, suggesting a correlation between tissue topology and higher-order processes in the SAM.

The connectivity of the cells in the epidermis of the plant hypocotyl, the embryonic stem tissue linking the above and below parts of the plant, was also examined using network science [11]. Two cell types are present in the hypocotyl epidermis, consisting of hair-producing cells (trichoblasts) and non-hair-producing cells (atrichoblasts) (figure 3*c*). While the genes which generate this pattern of alternating cell files have been well characterized, the functional significance of this configuration remains poorly understood.

Network analysis found the hair-forming trichoblast cells to have more neighbours (figure 3*d*) [11]. However, the non-hair-producing cells were found to lie upon more shorter paths (figure 3*e*). The capacity of each cell type to transport small fluorescent molecules was performed to explore the functional significance of this topological asymmetry. Non-hair cells transported more rapidly, as predicted based on lying upon shorter paths (figure 3*f*). This suggested the uncovering of a structure–function relationship in this tissue whereby hair cells are specialized for solute uptake, and their adjacent non-hair cells specialize in longitudinal solute transport based on their topological positioning within the multicellular system.

### 3.3. Cell differentiation patterns in the mouse embryo

One of the central questions of developmental biology is how different cell types can arise from a single cell during embryonic development. Network analyses of preimplantation mouse embryos combined the quantification of the tissue structure with analyses of the distribution of protein expression levels and cell fates to investigate the mechanisms underlying this cell differentiation process. The focus is on the blastocyst stage which occurs approximately from day 3.25 to day 4.5 of embryonic development [39]. At this stage, the embryo consists of the inner cell mass (ICM) which is surrounded by the trophectoderm that consists of placental precursor cells. The cells undergo a round of cell differentiation and give rise to the epiblast and primitive endoderm cells, the embryonic and yolk sac precursor cells, respectively [40].

In the network that represents the blastocyst, nodes represent cells and edges their physical contact [25,41]. The network and hence tissue structure was analysed by extracting the node degree. An ICM cell has approximately 30% more neighbours than cells from the surrounding trophectoderm (TE) [41]. Furthermore, the two cell types are clearly separated such that ICM cells have a majority of ICM neighbours and TE cells have mainly TE neighbours.

To analyse the cell differentiation patterns, the network was extended by integrating protein expression levels as node properties. The transcription factors NANOG and GATA6 are early markers for epiblast and primitive endoderm fate in the inner cell mass [42]. The extended network was used to determine correlations between a given cell and its neighbourhood in terms of protein expression. In an early stage of the mouse blastocyst, a local pattern arises in the inner cell mass that evolves into a global spatial segregation of epiblast and primitive endoderm [25]. The local pattern is characterized by a correlation of number of neighbours and NANOG expression of a cell as well as correlation between GATA6 expression between neighbouring cells in primitive endoderm precursors (figure 3*g*). These results suggest that the three-dimensional cell neighbourhood plays a role in epiblast and primitive endoderm specification.

For the comparison of *in vitro* models with the *in vivo* situation, network analysis provides a quantitative approach that exceeds mere visual inspection. In this context, the neighbourhood distributions of ICM organoids, as a three-dimensional *in vitro* model of the mouse ICM have been analysed [43]. ICM organoids do not contain TE cells and have 100 times as many cells as mouse ICMs. Nonetheless, the results show a good agreement between the *in vitro* and the *in vivo* system. This hints at an independence of the local cell fate patterns on the global tissue geometry and the number of cells.

### 3.4. Cancer tissue

Cancer is a developmental disease. Loss of cellular coherence leads to tissue dysfunction and hence loss of organism fitness. For studying cancerous tissue, network analysis provides a quantitative description of cancer cell clusters. Histological stainings allow visualizing the tumour cell distribution (figure 3*f*). For classical Hodgkin lymphoma, the tumour cells do not form a coherent structure but are sparsely distributed within the lymph node. Only 1–2% of the tissue is covered with tumour cells [26]. The images are transformed into tumour cell graphs, where nodes represent cells and edges indicate potential interactions via motile molecules [44]. Comparing the shape profiles of the node degree distribution in the network with the expected probability distributions shows that tumour cells cluster in the lymph node. Furthermore, adding shape information as node properties to the network reveals differential preferences for neighbourhoods of differently shaped cells (figure 3*f*) [26]. Interestingly, both the spatial distribution of the tumour cells and the neighbourhood distributions of particular cell shapes vary between subtypes of Hodgkin lymphoma.

For the analysis of breast tissue, the graph structure has been chosen to represent the lobular structure of the tissue [45]. Therefore, hierarchical graphs have been implemented [46]. In this case, a vertex does not represent a cell but a small cluster of cells. Edges are introduced based on the distance between two vertices. Apart from the spatial distribution of the cells, the features of the surrounding extracellular matrix (ECM) are important for tissue maintenance and cancer progression. ECM-aware cell graphs have been used to classify bone tissue into healthy, fractured and cancerous [45]. In these graphs, every vertex has a position and a colour representing the location of a cell and the composition of the surrounding extracellular matrix. Edges are included between two vertices that are close enough and that have the same colour.

In all cases, the network measures provide objective parameters for the structure of the tissue. Even though the relevance of these measures for automated tissue classification has been reduced by the emergence of convolutional neural networks in digital pathology, they are still of immense value for improving our understanding of the underlying biology.

### 3.5. Networks of cell processes in brain and bone

In animal, plant and human tissues, cells are densely packed. In this case, tissue topology can be described by defining cells as nodes that are connected to their direct neighbour cells via edges. By contrast, neurons in the brain have long processes that branch out and connect to distant cells via synapses. As a result, the number of neighbours to which a neuron can connect is not limited by cell packing.

In many tissues, the space between cells is filled with extracellular matrix. This intercellular space can also contain cell processes and cell–cell connections. For example, the mineralized bone matrix of most vertebrates contains a dense network of cells. It can be compared to brain tissue in terms of size and complexity [47]. The osteocytes that make up this network are differentiated bone cells that get encased in the mineral matrix and maintain contact with other bone cells and with blood vessels through a network of fine canals. This lacuno-canalicular network (LCN) forms during bone growth or regeneration and remains in place after mineralization, sometimes for decades [48]. Identifying cells with nodes, as in the examples above, does not capture the complexity of the canalicular network. Instead, all branching points of cell processes can be defined as nodes, and the canaliculi connecting them as edges [12].

Topological comparison of the resulting network in different types of bone tissue shows that the LCN in lamellar bone, which grows more slowly but in a more controlled manner compared with woven bone, is less densely connected, but more optimized for efficient transport. Nodes of high betweenness centrality are aligned along paths that connect different lamellae (figure 3*h*). Cumulative distributions of edge degree and link length are exponential, indicative of spatially constrained networks, and do not differ between bone types, suggesting conserved local biological growth rules. Differences in local connectivity, however, show that woven bone is more disordered, while the ratio of shortest path length to clustering coefficient of the LCN scales with network size in the same way as in other small-world biological networks [17]. Furthermore, the subcellular topology of the osteocyte LCN is highly relevant for the mechanosensory function of bone tissue, as it defines how fluid flows through the network of cavities [49].

## 4. Developmental rules underpinning the creation of cellular topology

**Figure 4.**
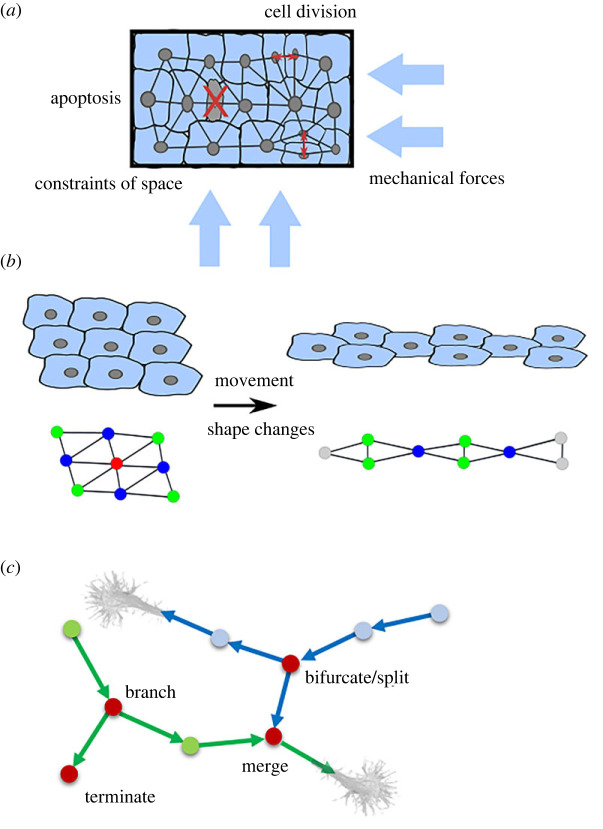
Illustration of developmental rules. (*a*) Rules that influence cell packing within a constraint tissue geometry. (*b*) Topological rearrangements due to cellular movement and cell shape changes. (*c*) Emergence of neuronal or osteocyte topology by growth cones splitting, merging and terminating.

In the following section, we aim to integrate the results from network analyses of diverse biological tissues by discussing the common generative principles that give rise to the different observed cellular topologies. In general, cell topology in tissues is generated through a combination of (i) the manner by which cells divide, (ii) changes to their shape relative to one another following division or due to mechanical forces and spatial constraints, and (iii) cellular movement and rearrangement within tissues (figure 4*a*). We consider each of these features separately below.

### 4.1. Cell division rules

Simple rules describing the symmetric division of cells in plant and animal tissue have been described [50]. A symmetric division takes place when the resulting sizes of the daughter cells are equal. The ‘default’ rules in both cases state that the cells will divide through the middle of the cell using the shortest possible division plane. In each *Drosophila* wing disc (Hoffmeister's rule), and in plant tissues (Errerea's rule), this default cell division rule was identified, whereby the shortest wall division plane passes through the centre and results in two equally sized progeny cells.

This division rule was expanded to consider the topological state of neighbours in the study mentioned above examining the number of neighbours in the *Drosophila* wing disc [8], representing a local topological cell division rule. A study examining the higher-order organization of cells in the plant SAM further extended a cell division rule leading to an emergent global topological property of maximized path length, i.e. no shortcuts through the tissue [34]. This arose by mechanical feedbacks between cells, impacting cell shape and setting up a shortest wall division plane (Errera's rule), which conforms to the global maximized path-length property. Such feedback between cell shape and a maximized global path length provides a link between local division rules and global emergent properties of a system.

In instances where cells are fixed in place with respect to one another, symmetric divisions are incapable of generating a pattern beyond ordered lattices. In order to create novel patterns, asymmetric divisions must occur, whereby the daughter cells are not of equal size. While the mechanisms underpinning the regulation of unequal cell division remain unknown, one factor proposed to play a role is that of mechanical force. A cell division rule has been proposed whereby cells divide perpendicular to the axis upon which mechanical force is applied to them. The mechanically responsive cell division rule is able to override the shortest wall cell division rule. Such oriented asymmetric divisions give rise to complex plant structures, such as the modification of leaves into pitcher plants [51].

### 4.2. Cell shape changes

Besides cell division, changes of cell shape due to growth, mechanical forces or spatial constraints can change the topology of a tissue. Mechanically guided cell growth can couple local control mechanisms to global tissue structure and topology [52]. The question if stress (force) or strain (deformation) are the driving mechanism may depend on whether a tissue is predominantly serially or parallelly connected [53]. In the *Arabidopsis* shoot apical meristem, local topology correlates with turgor pressure rather than cell wall mechanical properties, suggesting an active role of pressure for tissue topology and patterning since cell growth in plants is physically controlled by turgor pressure [54]. Also in many different epithelial tissues the distribution of node degree is related to the distribution of cell size [8]. A combination of experimental as well as computational perturbations of cortical contractility, line tension and resting area (cell size) revealed that physical constraints resulting from the reciprocal force balance between all cells determine the resulting tissue topology.

Cells in epithelia usually adopt a hexagonal surface-area minimizing shape. One exception is the irregular shape of epidermal pavement cells of plant leaves that results from their cell wall structure. The topology of the leaf epidermis is distinct from that of epithelia [55] and violates the Lewis' law that states that the number of neighbours is proportional to cell size, indicating that rather than area-filling constraints, other biophysical mechanisms play a role [56]. The observed leaf cell topology can be explained by a topology-driven cell division rule regulated by cell shape mechanics [55].

Geometric constraints can influence what shapes a cell can adopt. The topology of epithelia is usually studied on the apical surface of the tissue. In many cases, however, epithelial tissues are not flat, but curved. The resulting geometric constraints force cells to intercalate between the apical and basal surface [57], leading to different degree distributions on both sides of the tissue. Cells adopt complex ‘scutoid’ shapes with additional vertices between the apical and basal side to balance forces and satisfy mechanical energy minimization in the tissue. This shows that cell connectivity is tightly linked to tissue geometry via mechanical forces, and that tissue architecture can only be accurately captured by taking the full three-dimensional architecture into account, even in epithelia.

### 4.3. Cell movement and rearrangement

In densely packed tissues such as epithelia, topology is ultimately limited by the number of direct neighbours. To overcome this limitation, cells can form long protrusions that grow outwards to connect to more distant cells far beyond their direct neighbours. Examples include the axon and dendrites of neurons, osteocyte protrusions in bone, or nanotube-like processes in tendon cells [61]. The outgrowth of protrusions and the formation of connections with other cells determine the resulting topology, while the initial cell packing becomes less relevant in this case. Mechanisms of protrusion outgrowth are linked to cell migration and involve cell adhesion and actin polymerization [58]. These processes are guided by various environmental cues, including matrix rigidity, anisotropy, growth factors and even electromagnetic fields [52,53,59].

The physical state of tissues has previously been proposed to follow different states of fluidity, ranging from liquid to solid [60]. In multicellular systems, where cells move with respect to one another, topological reconfigurations can occur readily, and represent ‘liquid’ systems (figure 4*b*). In other cases, such as in plant tissue, cells are fixed in position with respect to one another, making their topologies fixed, and represent a ‘solid’ system. Cell slippage has not been reported in plants. The physical state of the cells in a tissue reflects the mechanical properties of the biological system. Cell adhesion is more flexible in fluid systems and more stringent in the case of solid systems.

## 5. Generative models for tissue network development

Understanding how iterations of the local developmental rules outlined above give rise to observed topological properties in tissues is often not straightforward. Computational models that generate tissue topology based on hypotheses for the developmental rules have been pivotal in different developmental systems. Depending on the scientific question, models of different complexity are appropriate. In this section and in table 1, we give an overview of the different modelling approaches used to study the link between local rules and the resulting global topology. We start with the least complex.

**Table 1.** Summary of the biological patterning process studied using generative models.

model type	biological question	mechanism	modelling approach	topological measure	ref.
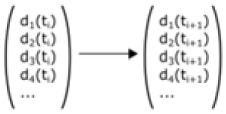	epithelial packing in *Drosophila, Xenopus, Hydra*	cell division	Markov model for number of neighbours, no spatial component, rule based cell division	degree	[8,62]
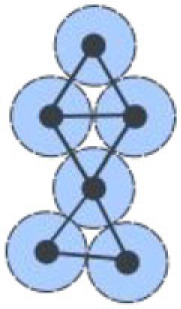	epithelial packing in *Drosophila*	cell division, mechanics and boundary conditions	two-dimensional centroid model, cell cycle model, mechanics	degree of all cells, degree of mitotic cells	[63]
	global connectivity in plant apical meristem and virtual tissue	cell division rules	cell division rules in a three-dimensional centroid model using local geometric and global topological rules	degree distribution and path length	[34]
	cell fate patterning in ICM organoids	random fate allocation and cell division	cell division rules in a three-dimensional centroid model	local cell fate distribution	[64]
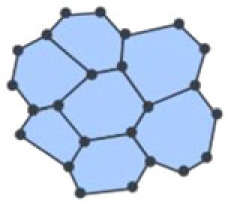	epithelial packing in *Drosophila* wing disc	cell mechanics, cell division	cell mechanics and symmetric divisions in a two-dimensional vertex model	degree	[32]
	local epidermal connectivity in virtual two-dimensional tissue and plant tissue	cell division and simulation of mechanical forces	vertex model with cell division following different division rules	degree distribution	[65]
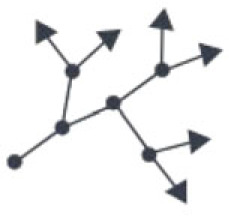	dendritic cell connectivity during neural tissue development	outgrowth, branching and merging, mechanical interactions	hybrid grid/continuous three-dimensional	in-degree, shortest path length, count of motifs	[66,67]
	dendritic cell connectivity during neural tissue development	outgrowth, merging, branching of cell processes	continuous two-dimensional/three-dimensional	in-degree, shortest path length, motifs, small worldness	[66,68]
	dendritic cell connectivity during osteocyte network formation	dendrite growth, bone deposition	mean field two-dimensional	degree	[69]
	dendritic cell connectivity during osteocyte network formation	outgrowth, merging, branching, environment	hybrid grid/continuous three-dimensional	degree, edge length, centralities, small worldness	[70]

### 5.1. Markov models

A discrete Markov chain model describes the evolution of a system as a series of states in which the probability of transitioning to the next state only depends on the current state [71]. In the case of tissue networks, the state of a tissue has been described as a vector of frequencies of node degrees [8,62] (table 1). Hence, an entry in the vector contains the frequency of cells with a specific number of neighbours. Proliferation rules specify the probability of an i-sided mother cell to produce a j-sided daughter cell and thereby determine the probability for the system of transitioning from one state to the next. The spatial arrangement of the cells in the tissue context or any mechanical interactions are not considered. Such a model reproduces the empirically derived distribution of node degrees in different epithelia [8,62] (table 1). These results propose that cell proliferation dynamics on their own are sufficient to cover the complex topological situation in epithelia.

### 5.2. Agent-based models

Agent-based models represent complex systems by considering the individual constituents [72]. They comprise a collection of autonomous agents that behave according to a predefined set of rules. The rules can be discrete, such as if-else statements, or continuous, based on differential equations. In tissue networks, the individual nodes are the agents and their behaviour depends on rules for the individuals as well as interaction rules for nodes connected by edges. A key distinction between agent-based models is how much spatial detail is implemented. Two types that have been applied to tissue networks in several contexts are the centroid and the vertex model.

#### 5.2.1. Centroid model

The agents are cells that are assumed to be discs in two dimensions or spheres in three dimensions and are described by their position in space and an interaction radius [73]. Hence, the set-up is a tissue network with nodes that have the position as attributes and edges that occur if two cells are closer than the sum of their interaction radii. The position of a cell changes due to mechanical cell–cell interactions if two cells are connected by an edge. Additionally, rules for cell motion, cell division and apoptosis can be implemented. In a more refined version, the edges that define cell–cell interaction are included based on a Delaunay triangulation [64]. Often, the centroid model is combined with a Voronoi tessellation [34,72]. These allow polygonal approximations of the shape of cells.

The application of a centroid model as a generative model allows testing different parameters that affect tissue topology individually. The effect of the orientation of the division plane, the duration of the cell cycle, the mechanical properties of the cells as well as boundary conditions on the node degree of a tissue have been analysed with a centroid model [63]. Comparison with experimental data for *Drosophila* epithelia allowed determining suitable parameter value combinations.

A different approach for analysing the effect of a parameter of interest is neglecting it in a model. Jackson *et al.* generated multicellular assemblies based on three-dimensional anisotropic Voronoi tessellation that resembled the *Arabidopsis* SAM in terms of node degree and random walk centrality but not with respect to the cell size distributions [34]. Applying a set of division rules to this virtual tissue showed a different behaviour to that observed in experiments. Hence, the correct cell geometry is essential, which has also been verified experimentally by mutant analyses.

Apart from the basic tissue topology, the distribution of cell fates is also of major interest in developmental systems. In a centroid model, cell fate markers can be added as properties to each cell. In this case, cell division rules determine how a fate is passed from the mother cell to the daughter cells. Testing different inheritance rules in a centroid model and subsequent comparison with experimental data revealed that symmetrically passing on the same fate from mother to daughters best reproduces the cell fate patterns during primitive endoderm differentiation in ICM organoids [64].

#### 5.2.2. Vertex model

A vertex model allows to introduce more detailed geometric features of the cell shape than the centroid model [32]. Each cell is represented as a polygon. This corresponds to a tissue network in which the nodes describe the polygon corners and the edges the polygon sides. Forces acting on a cell encompass cell elasticity and adhesion. Those are described by area elasticity, line tension at junctions between cells and contractility of the cell perimeter. Cellular packing is determined by stationary and stable network configurations that fulfil a force balance. Cell division is implemented as the introduction of a new edge based on a division centre and a division direction [74].

Such a vertex model has been used to study the packing geometry, in particular the node degree, in the *Drosophila* wing disc [32]. Interestingly, only a static tissue without proliferation can maintain a perfect hexagonal packing. Proliferation yields irregular cell shapes.

For plant epidermis, a diversity of cell division rules were capable of giving rise to observed degree distributions. When also considering geometrical properties, cell division rules which favoured equal-sized cells most closely approximated the observed data for node degree and internal vertex angle [65].

#### 5.2.3. Continuous models

For the study of dendritic cell connectivity, the cell–cell connections are of particular interest, while the spatial arrangement of the individual cells is less important. In these cases, a continuous model can be applied, implemented as the growth of a network by adding nodes and edges via branching and merging of growth cones.

The simulation tool NETMORPH implements neurite outgrowth and branching based on phenomenological rules [66,68]. The neurites can elongate and bifurcate with given probabilities. If two neurons are in close proximity, a synapse can form. The model has been validated with respect to rat cortical layer 2/3 pyramidal neurons. The simulation tool CX3D further considers the appropriate mechanical properties of individual neurons [66,67]. This continuous growth model is combined with diffusion of extracellular signalling molecules in the surrounding space. The model has been shown to generate cortical layers, different branching patterns based on extracellular signalling molecules and network formation in dissociated culture neurons.

Including spatial constraints into a continuous model of network growth increases its range of possible applications. Three-dimensional growth and branching have been described by a random walk that is biased by local environmental conditions and constraints [70]. The local environment is either simulated or can be extracted from microscopy images that show e.g. extracellular matrix organization or the distribution of soluble cues such as growth factors. Sensitivity analysis for the different modelling parameters such as branching probability or angle show the effect of the local growth parameters on the tissue topology. Different to most studies, in addition to the node degree, a number of further network measures such as clustering coefficient, shortest path, edge density, betweenness and centrality measures have been considered.

Focusing on the question of interest and the available experimental data when choosing the appropriate modelling approach is essential. Increasing model complexity always comes with increasing computation time but can only offer a knowledge gain if it is suitable for the respective question.

## 6. Discussion

The application of modelling approaches including the use of generative models enables the identification of plausible mechanisms which give rise to complex multicellular assemblies [73]. Functional tissue organization emerges from cell–cell interactions and external cues without centralized control [74]. This developmental process is an example for rule-based iterative generation of patterns. The local rules in this case correspond to how and when cells divide, migrate or differentiate. This set of rules is encoded in the genome, but the activation of specific genes or genetic programmes depends on chemical or physical signals received by the cell, defined by its connectivity to other cells in the local tissue environment. Tissue topology and architecture, in turn, depends on prior cell activity, creating a feedback loop in space and time. Reciprocity and feedback loops are a necessary component of stable control systems and enable robust control and homeostasis in the presence of noise and external perturbation [75]. Identifying these feedback loops is essential to understand how genotype and phenotype are related, and a key question in developmental biology. Tissues are networks of cells, and identifying the generative rules that determine their topology can help to understand the control principles behind tissue development.

Here, we collected existing work from different areas of biology where the mechanisms underlying tissue topology have been quantified and related to generative mechanisms. In the future, the link between generative rules (genetic programmes) and tissue topology independent of a specific model system could be identified by systematically comparing exemplary model systems. The cross-kingdom application of network-based approaches towards understanding tissue complexity may provide a means towards the identification of unifying principles underpinning multicellular life and evolutionary processes. Despite the biological diversity and independent evolution of multicellularity, the possibility that common physical principles underlie complex life remains. These may be related to structure–function relationships in cell organization [18], and their progressive optimization through selective forces. Understanding such principles may serve as guiding features in understanding the basis of complex life and its engineering. In the future, combinations of the modelling approaches described here with machine learning could make it possible to infer the rules and parameters of the generative models directly from experimental data, e.g. via simulation-based inference or automatic differentiation. This could make such models more predictive by connecting them to experimental image data and might even lead to applications in image-based diagnostics by detecting pathological changes at the cellular level based on macroscopic topological changes at the tissue level.

## Data Availability

This article has no additional data.
